# Emerging Viral Infections in Sub-Saharan Africa and the Developing Nervous System: A Mini Review

**DOI:** 10.3389/fneur.2018.00082

**Published:** 2018-02-23

**Authors:** Angelina Kakooza-Mwesige, Abdul H. Mohammed, Krister Kristensson, Sharon L. Juliano, Julius J. Lutwama

**Affiliations:** ^1^Department of Paediatrics and Child Health, Makerere University College of Health Sciences and Mulago Hospital, Kampala, Uganda; ^2^Department of Psychology, Linnaeus University, Växjö, Sweden; ^3^Department of Neuroscience, Karolinska Institutet, Stockholm, Sweden; ^4^Anatomy, Physiology and Genetics, Uniformed Services University of the Health Sciences, Bethesda, MD, United States; ^5^Arbovirology Laboratory, Uganda Virus Research Institute, Entebbe, Uganda

**Keywords:** developing nervous system, emerging viruses, Ebola virus, Chikungunya virus, West Nile virus, Zika virus, neurological disorders, sub-Saharan Africa

## Abstract

The global public health concern is heightened over the increasing number of emerging viruses, i.e., newly discovered or previously known that have expanded into new geographical zones. These viruses challenge the health-care systems in sub-Saharan Africa (SSA) countries from which several of them have originated and been transmitted by insects worldwide. Some of these viruses are neuroinvasive, but have been relatively neglected by neuroscientists. They may provide experiments by nature to give a time window for exposure to a new virus within sizeable, previously non-infected human populations, which, for instance, enables studies on potential long-term or late-onset effects on the developing nervous system. Here, we briefly summarize studies on the developing brain by West Nile, Zika, and Chikungunya viruses, which are mosquito-borne and have spread worldwide out of SSA. They can all be neuroinvasive, but their effects vary from malformations caused by prenatal infections to cognitive disturbances following perinatal or later infections. We also highlight Ebola virus, which can leave surviving children with psychiatric disturbances and cause persistent infections in the non-human primate brain. Greater awareness within the neuroscience community is needed to emphasize the menace evoked by these emerging viruses to the developing brain. In particular, frontline neuroscience research should include neuropediatric follow-up studies in the field on long-term or late-onset cognitive and behavior disturbances or neuropsychiatric disorders. Studies on pathogenetic mechanisms for viral-induced perturbations of brain maturation should be extended to the vulnerable periods when neurocircuit formations are at peaks during infancy and early childhood.

## Introduction

Exposure to infections during the first part of fetal life, the so-called teratogenic window, can cause severe brain malformations. To the established human neuroteratogenic pathogens (*Toxoplasma gondii*, Other pathogens, Rubella, Cytomegalovirus, and Herpes simplex virus; TORCH), Zika virus (ZIKV) is now added (1–3). Perinatal, infant, and childhood infections may also disturb the developing nervous system during the peaks of neurocircuit formations and possibly cause more subtle changes in brain maturation. Thus, cognitive impairments and behavioral disturbances in children born to HIV-infected mothers (4) or subjected to childhood malaria (5) have been described in SSA; unprovoked late-onset epilepsy may also occur following the latter infection (6).

Of particular concern to African neuroscience are emerging viral infections. They can reveal associations between infections and rare sequelae in human populations, as poliomyelitis once did for “infantile paralysis” (7). Early life viral infections may, in one way or the other, be implied in the pathogenesis of cognitive and neuropsychiatric disturbances: the “neurodevelopmental hypothesis” for late-onset brain dysfunctions [e.g., Ref. (8)]. By comparing four neuroinvasive infections originating from sub-Saharan Africa (SSA), i.e., ZIKV, West Nile virus (WNV), Chikungunya virus (CHIKV), and Ebola virus (EBOV), we find that they attack the human brain at various stages of development. Thus, time windows for viral invasions, given by occurrence of the emerging epidemics, may reveal unique associations between viral infections and late-onset human brain disturbances.

## Discovery of the Viruses and the Magnitude of the Problem

**Figure 1:**
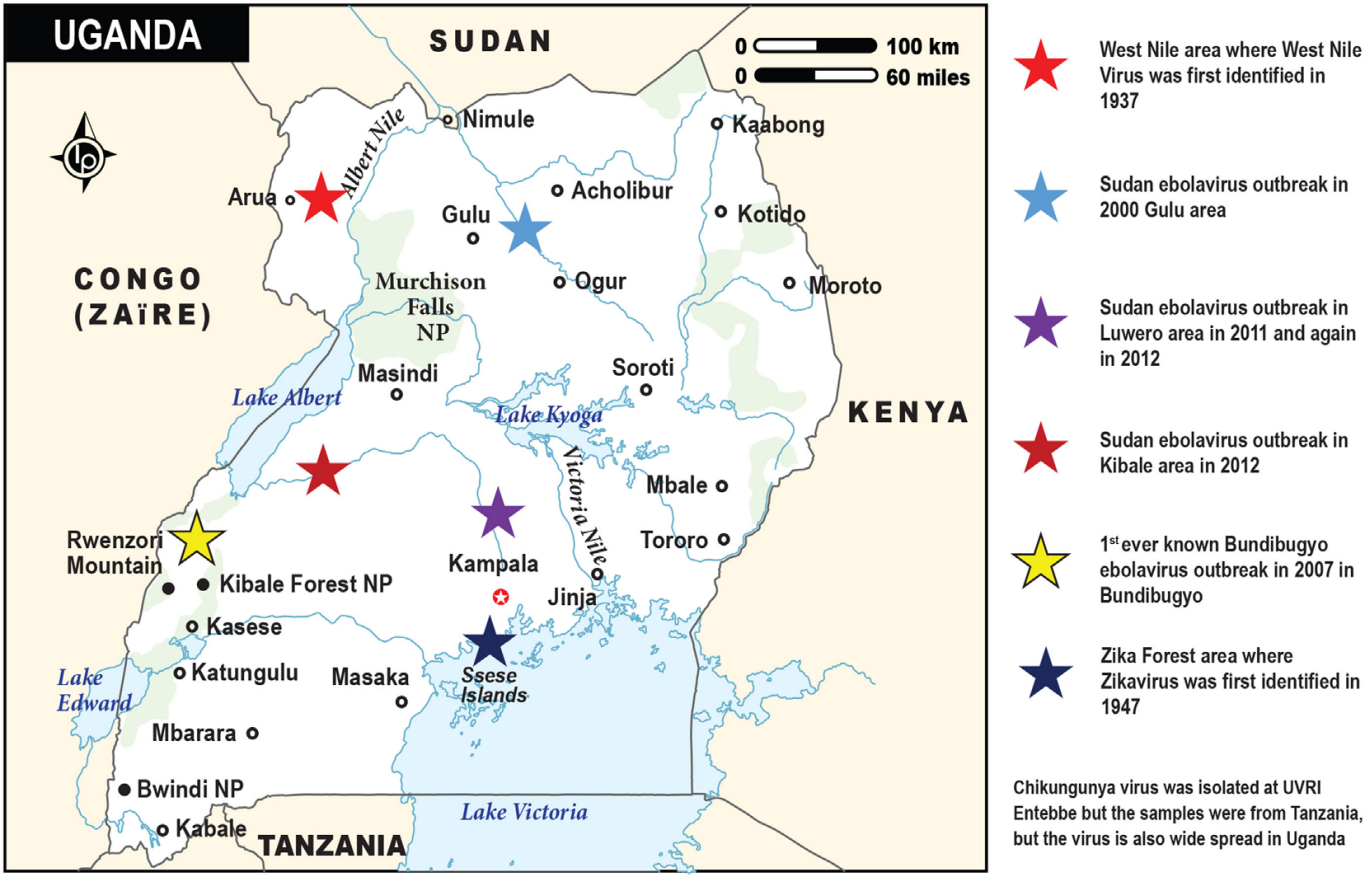
The map of Uganda showing the sites and times for the discoveries of the emerging viral infections by Ebola, West Nile, and Zika viruses. Chikungunya virus (not depicted on this map) is also widespread in Uganda. It was first isolated at the Uganda Virus Research Institute in Entebbe from blood samples obtained from Tanzania (Tanganyika territory). The various viral outbreaks are denoted by the stars in different colors, while the respective country locations are indicated by the small black circles.

The emerging viruses dealt with in this review are indigenous to and were first identified in Africa (Figure 1). WNV was isolated from a woman, who had a mild febrile illness in the West Nile Region of Uganda in 1937 (9). ZIKV was first isolated from a Rhesus monkey, placed on a platform as bait for mosquitoes in studies on yellow fever in the Zika forest, in Uganda in 1947 (10). CHIKV was first isolated in 1953 at the Uganda Virus Research Institute from samples collected in Tanganyika (11). In 1976, the investigation of concurrent outbreaks of a hemorrhagic fever syndrome in Zaire (currently Democratic Republic of Congo) and Sudan (currently Republic of South Sudan) led to isolation of two viruses now referred to as EBOV and Sudan virus, respectively. Another member of the EBOV, Bundibugyo Virus was identified in Uganda (12) (Figure 1).

All these viruses, which are endemic in tropical SSA, have had outbreaks also in other African regions, e.g., WNV in north and west Africa, in South Africa, and in Madagascar during the last decade (13). Currently, there is an outbreak of CHIKV in Kenya. The mosquito-borne WNV, ZIKV, and CHIKV have spread worldwide out of Africa. For instance, WNV is the most common cause of encephalitis in the US, enhanced by the bird reservoir hosts (14). CHIKV has caused extensive epidemics on islands in the Indian Ocean, in particular, La Reunion (15–17). The spread of these emerging viruses into previously unaffected regions can be attributed to more frequent and distant travels, evolution of mutant viral strains with altered virulence, and changes in climate and local ecosystems.

## Sensory Cues in Mosquito–Human Interaction

Neuroscientific research is important to design specific, repellant molecules that reduce mosquito’s attraction to humans. Three of the emerging infections discussed are mosquito-borne, i.e., *Aedes aegypti* for CHIKV and ZIKV and *Culex pipiens* for WNV; *Aedes albopictus* can also host ZIKV (18). Neurophysiological and behavioral studies have revealed *A. aegypti* attraction cues emanating from humans [e.g., Ref. (19)]. Odor is one important cue of insect host-seeking behavior [e.g., Ref. (20)]. The mosquito’s olfactory receptor neurons can detect various odor volatiles from the human host, which make some people more attractive than others to mosquitoes. Carbon dioxide (21), lactic acid (22), and visual cues (23, 24) can also attract mosquitoes to the host. Neuroanatomical studies have uncovered the organizations of olfactory centers in the mosquito brain (25), and multimodal integration of carbon dioxide, body odor, and temperature cues in the mosquito brain can enhance mosquito attraction to humans (26). Novel strategies that specifically block more than one sensory cue for attraction of insects are envisioned to contribute to control of emerging viral spread and outbreaks.

## Entry Routes for Viruses to the Brain: Effects of Neuroinvasive Viral Infections During Development

Following the sting of an infected insect, neurotropic viruses enter through the skin, replicate in susceptible cells, and spread to the central nervous system (CNS) *via* peripheral nerve fibers and/or the bloodstream (27). WNV can directly spread by the former route (28), while viral spread *via* the bloodstream into the brain is hampered by barriers, i.e., the blood–CSF barrier and the blood–brain barrier (BBB), established early during embryonic life (29). Nevertheless, certain viruses pass across these barriers and target neural cells. Of the presently described viruses, the BBB may be crossed by WNV (14), ZIKV (see below), and probably also EBOV (30), while CHIKV can pass the blood–CSF barriers through the permeable choroid plexus to infect ependymal cells in a mouse model (31).

During pregnancy, viruses can enter the fetal brain through the bloodstream following placental transmission. Importantly, the placental structure as well as its expression of receptors used by various viruses and its innate immune response differ markedly between humans, mice, and ruminants, which hinders direct comparisons among animal species (3). Notably, human fetal immune adaptations provide early postnatal protection against extracellular pathogens but enhance the risk of virus-induced persistent infections (32).

**Figure 2:**
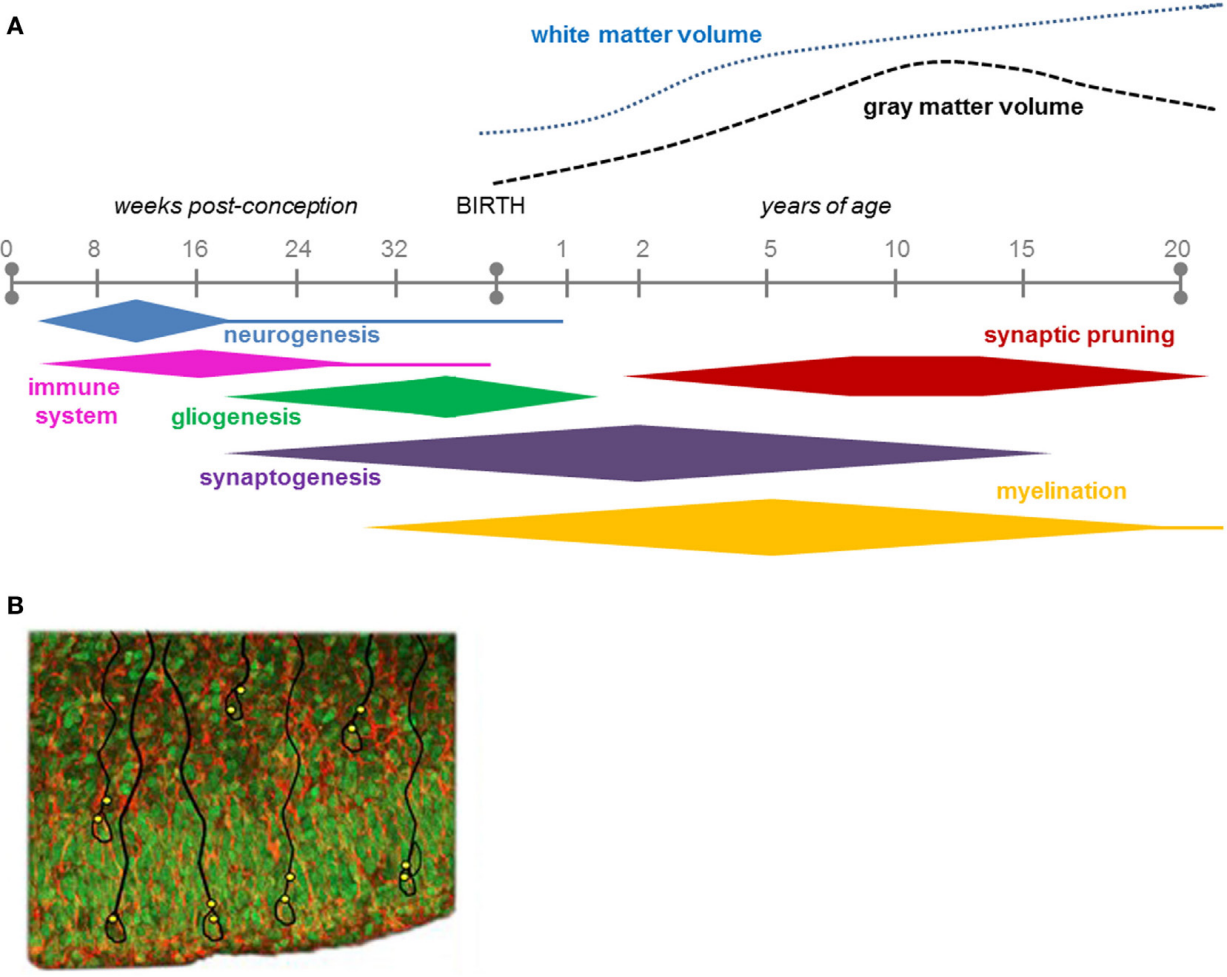
**(A)** The time course of key neurodevelopmental processes in humans, during gestation and up to 20 years of age (29). Note that most of the neurogenesis is completed before birth, while most of the synaptogenesis continues after birth into adolescence. Figure 2A is reproduced with permission from Elsevier. **(B)** Ventricular zone and subventricular zone showing numerous proliferative cells (green; Sox2 immunolabeling) that will populate the developing cerebral cortex. They form the nuclei of radial glial cells (red/orange; vimentin) some of which are outlined in black. Indicated in yellow are presumptive sites of entry of the Zika virus, which appears to specifically attack neural progenitor cells to cause microcephaly.

The effects of an intrauterine infection depend on timing of the insult with highest vulnerability during peaks in neuronal cell proliferation and migration (33). In humans, simplified gyral patterns may result from disturbed cell migration at 8–16 weeks of gestation. While congenital microcephaly (reduced head circumference) is associated with various insults causing disturbed proliferation, migration, or destruction of cells at various periods of intrauterine life (34). Since the human brain increases about 3.5 times in weight after birth, when neuronal network formation and myelination are at their peaks, the question can be posed whether infant/childhood infections may cause less conspicuous, functional disturbances of the developing nervous system than malformations as long-term or late-onset effects [Figure 2A; (29)]. In fact, a register-based study has indicated that children with severe viral CNS infections at 0.5–8 years of age show enhanced risk of psychotic illness when they reach young adulthood (35).

## Clinical Effects of the Emerging Viral Infections on the Developing Nervous System

### Zika Virus

Zika virus infections have been endemic for decades in SSA (36), but effects on the brain were not reported until the virus spread out of Africa. The infection is most often asymptomatic or associated with mild signs of disease such as maculopapular rash and non-purulent conjunctivitis (37). Ultrasonographic studies in Brazil of ZIKV-infected pregnant women carrying fetuses diagnosed with microcephaly, showed in addition, severe progressive ventriculomegaly, periventricular and basal ganglia calcifications, corpus callosal dysgenesis as well as posterior fossa abnormalities [e.g., Refs. (38, 39)]. Neuropathological findings in 11 newborns with congenital microcephaly include small brains with almost complete agyria, neuronal heterotopia, enlarged ventricles with or without aqueduct stenosis, and well-formed brains with calcifications (40, 41). Only two cases of perinatal infections, discharged in good health, and a limited number of children infected *via* mosquitoes, have been reported; they were mildly infected or asymptomatic-like adults (42).

Recent neuroimaging reports of infants with postnatal microcephaly or normal skull size, but with other signs of congenital Zika syndrome, indicate a spectrum with less severe brain changes (43, 44). Whether the virus can persist in the human infant brain during postnatal development remains to be clarified. Related to this, a critical question is whether disturbances in cognitive development may appear later in life even following asymptomatic infections in newborn, which may occur in the majority of children borne to ZIKV-infected mothers [cf. Ref. (45)]. Currently, no information is available on ZIKV-related nervous system disturbances in SSA.

### West Nile Virus

Most WNV infections are asymptomatic, but some are associated with flu-like illness and maculopapular rash. Less than 1% are neuroinvasive causing meningitis and encephalitis, which increases in incidence by age (14, 46); flaccid paralysis, movement disorders with tremor, and myoclonus are other rare complications (47). Adult patients recovering from WNV encephalitis can show low scoring in cognitive and other neuropsychological tests for various periods of time (48, 49).

Studies of effects of maternal WNV infections during pregnancy on early childhood development in SSA do not exist. Studies from the US initially indicated a risk of abnormalities such as microcephaly, while two later retrospective and prospective studies showed no signs of CNS malformations or short-term development disturbances when compared to controls (50, 51). However, the number of children in these studies was small (50), and the incidence of neuroinvasive WNV infections in children is probably underestimated (52, 53). More studies are essential including long-term follow-up to assess potential effects of neuroinvasive WNV disease during various neurodevelopmental vulnerable periods (54). For instance, it would be of interest to conduct research into disturbances in the development of cognitive functions in the reported several hundreds of children of different ages surviving this disease since 1999 (55).

### Chikungunya Virus

In contrast to the previous two arboviruses, the majority of CHIKV-infected individuals develop symptoms such as fever and arthralgia. Meningoencephalitis and fatal outcome are rare. Mother-to-child transmission of CHIKV is also a rare event that has been reported in large-scale outbreaks of the disease such as on La Reunion 2005–2006. Although maternal infections long before delivery showed no observable effects on the outcome and there is no evidence of congenital virus transmission (56), perinatal infections with nervous system involvement do occur (57). Of 30 neonates with acute neurological manifestations, 2 died and 5 showed abnormal MRI scans (high intensity in periventricular white matter and corpus callosum). Five had neurological sequelae at discharge and 6 months later. These sequelae included behavior and communication disorders, autism and echolalia, recurrent seizures; one child had microcephaly and strabismus (58). In another follow-up study from the island about 50% of perinatally infected children showed at 2 years of age delay in development of coordination and language skills as well as in sociability and movement performance. Five out of 12 newborns with neonatal encephalopathy developed postnatal microcephaly with severe reduction of the white matter visualized on MRI (59). More long-term consequences of these perinatal and childhood infections remain to be studied.

### Ebola Virus

Ebola virus, which has bats as intermediate hosts (60), causes rapidly progressive severe hemorrhagic fever with a very high lethality rate. Based on findings from Ebola virus disease (EVD) studies done in West Africa, complications occurring >10 days from disease onset include meningoencephalitis (61, 62). The particularly vulnerable patient populations include children <5 years of age, the elderly, and pregnant women.

Systematic longitudinal assessments of EVD survivors in Africa are scarce. However, new insights and understanding about the long-term effects of infection are currently being generated from the survivors of the largest ever epidemic of EVD to date that occurred in Guinea, Liberia, and Sierra Leone (63). Interim analysis of data from this multidisciplinary longitudinal study of 804 EVD survivors in Guinea (20% are children) reports that EVD survivors exhibit an array of neurological and psychiatric symptoms even after more than 1 year following discharge from the hospital (64). The majority of survivors experienced physical disorders such as psychosocial problems, depression, or ophthalmological problems.

Given the paucity of current data, there is need for systematic longitudinal assessments of EVD survivors to clarify the spectra of nervous system sequelae and the magnitude of the problem. Of paramount interest in this respect is that EVD can persist after recovery in the brain of non-human primates (65).

## Obtaining an Experimental Model of ZIKV and the Developing Nervous System

Spurred by the great awareness of ZIKV-induced microcephaly, data on molecular mechanisms underlying this teratogenic virus infection are now rapidly accumulating. Here, we briefly review some recent findings and indicate gaps-in-knowledge for neuroscience to fill not only for teratogenesis but also for potential postnatal developmental disturbances in this and the other emerging viral infections.

Nowakowski et al. (66) suggested that AXL could be a candidate as a receptor for endocytotic entry of ZIKV into the fetal brain. AXL is a member of the multifunctional TAM receptor protein tyrosine kinase family that among other things regulates phagocytosis and plays a pivotal role in innate immune responses (67). In the human fetal brain, AXL is expressed in neural stem cells that generate neurons populating the neocortex, radial glial cells, astrocytes, and endothelial cells [Figure 2B; (66)]. From an infected mother, ZIKV may have a privileged entry to the placenta as indicated by the finding that it infects human endothelial cells of the umbilical vein in culture by binding to AXL with a high efficiency compared to other flaviviruses, such as WNV (68). Additional evidence suggests that the ZIKV infects other cells of the placenta, e.g., macrophages and cytotrophoblasts (69). Chavali et al. (70) recently demonstrated that the ZIKV has a predilection in its RNA genome to bind to a specific RNA-binding protein (Musashi-1), which is liberally expressed in the radial glial cells. Since these progenitor cells are precursors for neurons and astrocytes that form the cerebral cortex (71), this binding capacity may directly influence formation of the cerebral cortex.

Additional experiments using various types of cell culture have been useful in furthering our knowledge of ZIKV infection, especially on neural progenitor cells. Direct infection of human tissue placed in culture shows special susceptibility of radial glial and epithelial stem cells, while demonstrating limited direct infection of neurons (72). Since human progenitor cells form three-dimensional organoids in culture, they demonstrate features of radial glial cells, which also show evidence of specific direct infection in culture in this model. Numerous other experiments using various cell culture models and direct infection of vertebrates also demonstrate involvement of radial glial-like cells and progenitor cells [for review, see Ref. (73)]. This apparent focus of infection specificity to neural progenitor cells may help to clarify the preferential effect of ZIKV on the fetus leading to microcephaly.

Development of animal models is clearly an essential route to more completely understand ZIKV effects on the brain and the nervous system. Several such models exist to study effects of ZIKV infections in offspring to pregnant animals, but none of them fully replicate the human situation [for review, see Ref. (74)]. Viruses mostly need adaptations to a new animal species, the neurovirulence between viral strains differs, the placental immune response and receptors that viruses use differ between species (see, above), and even within the same species, such as mice, various strains differ markedly between their reactions to an infection. Nonetheless, offspring to ZIKV-infected immune-deficient mice and non-human primates have shown some abnormalities in the brains (74). Considerable efforts are needed for neuroscience–microbiology collaborations to develop suitable animal models of viral infections to disclose and validate not only neuroteratogenic viral effects but also more subtle alterations that may result from prenatal abortive or persistent infections.

## Conclusion and Perspectives

We present evidence that various spectra of neurodevelopmental disturbances may be associated with the emerging viral infections: ZIKV with congenital microcephaly following intrauterine infections, but also postnatal brain alterations with or without microcephaly; CHIKV with postnatal microcephaly following perinatal infections, but also behavior and communication disturbances; WNV with cognitive dysfunctions following infections mostly in adults, but infant/childhood infections are underestimated and need further studies. EVD, which has flared up in waves in SSA, may also be a threat to the developing human nervous system. Important gaps in knowledge include risks of cognitive impairment, behavior disturbances, and neuropsychiatric diseases following infections during infancy and childhood with these emerging infections, when neuronal network formation and synaptogenesis are at the peak. The molecular mechanisms resulting in disrupted neural development need to be unraveled in more detail and the possibility addressed whether some of these emerging viruses can persist in the human brain.

Based on this situation, we foresee a number of activities to reduce the menace of these infections to nervous system maturation:

- Strengthening capacity of African virology and neuroscience research with enhanced worldwide collaboration to strongly benefit the community.
- Development of human pluripotent stem cell models to use for screening of neuroinvasiveness along with other animal models that allow studying the timing and selectivity of infection as well as the pathogenetic and pathophysiological mechanisms of brain developmental disturbances.
- Large-scale epidemiological studies in SSA that determine not only the immediate effects on neurodevelopment parameters after early life exposure to emerging viral infections but also long-term consequences resulting in late-onset nervous system disorders.
- Development of innovative tools to prevent vector–host interactions that control the spread of viruses. Examples include the use of wearables, sprays, and skin lotions that block a selected repertoire of sensory mosquito receptors.

## Author Contributions

The authors contributed expertise information and presented one section each at a symposium with the same title at the SONA conference in Entebbe, Uganda, June 11–14, 2017. All the authors approved the final version of the manuscript.

## Conflict of Interest Statement

The authors declare that the writing of this article was conducted in the absence of any commercial or financial relationships that could be construed as a potential conflict of interest.

## Abbreviations

CHIKV, Chikungunya virus; CNS, central nervous system; CSF, cerebrospinal fluid; EVD, Ebola virus disease; SSA, sub-Saharan Africa; WNV, West Nile virus; ZIKV, *Zika* virus.
